# Prevalence of Anxiety in University Students during the COVID-19 Pandemic: A Systematic Review

**DOI:** 10.3390/ijerph19010062

**Published:** 2021-12-22

**Authors:** Shefali Liyanage, Kiran Saqib, Amber Fozia Khan, Tijhiana Rose Thobani, Wang-Choi Tang, Cameron B. Chiarot, Bara’ Abdallah AlShurman, Zahid Ahmad Butt

**Affiliations:** School of Public Health Sciences, University of Waterloo, Waterloo, ON N2L 3G1, Canada; siliyanage@uwaterloo.ca (S.L.); k2saqib@uwaterloo.ca (K.S.); af6khan@uwaterloo.ca (A.F.K.); trthoban@uwaterloo.ca (T.R.T.); wc4tang@uwaterloo.ca (W.-C.T.); cbchiarot@uwaterloo.ca (C.B.C.); baalshurman@uwaterloo.ca (B.A.A.)

**Keywords:** university students, COVID-19, anxiety, stress, mental health

## Abstract

There is a dearth of evidence synthesis on the prevalence of anxiety among university students even though the risk of psychological disorders among this population is quite high. We conducted a quantitative systematic review to estimate the global prevalence of anxiety among university students during the COVID-19 pandemic. A systematic search for cross-sectional studies on PubMed, Scopus, and PsycINFO, using PRISMA guidelines, was conducted from September 2020 to February 2021. A total of 36 studies were included, using a random-effects model to calculate the pooled proportion of anxiety. A meta-analysis of the prevalence estimate of anxiety yielded a summary prevalence of 41% (95% CI = 0.34–0.49), with statistically significant evidence of between-study heterogeneity (Q = 80801.97, I^2^ = 100%, *p* ≤ 0.0001). A subgroup analysis reported anxiety prevalence in Asia as 33% (95% CI:0.25–0.43), the prevalence of anxiety in Europe as 51% (95% CI: 0.44–0.59), and the highest prevalence of anxiety in the USA as 56% (95% CI: 0.44–0.67). A subgroup gender-based analysis reported the prevalence of anxiety in females as 43% (95% CI:0.29–0.58) compared to males with an anxiety prevalence of 39% (95% CI:0.29–0.50). University students seem to have a high prevalence of anxiety, indicating an increased mental health burden during this pandemic.

## 1. Introduction

COVID-19 was declared a public health emergency of international concern (PHEIC) by the World Health Organization (WHO, Geneva, Switzerland) [1] and as a pandemic on the 11th of March 2020 [2]. A pandemic is more than a medical phenomenon; it affects individuals and society and causes disruption, anxiety, stress, stigma, and xenophobia [3]. Public health emergencies, such as pandemics, take a toll on physical as well as mental health. The restrictive measures during the COVID-19 pandemic undoubtedly have affected the social and mental health of individuals from across the board [4]. It has created a psycho-emotional chaotic situation around the globe with an increase in mental health problems, including anxiety, depression, stress, sleep disorder, as well as fear [5,6], that eventually increased substance use [6] and sometimes suicidal ideation [7].

COVID-19 is a global concern affecting higher education institutions (HEIs) all around the world, and its impacts have been reported in universities across the world [8]. The sudden change due to the potential risk of death caused by COVID-19, isolation, and lockdown has increased the anxiety level and stress to the general public [9] and students [8,10]. Physically closing educational institutions (schools, universities) proved to be an efficient way of minimizing the spread of the virus, yet it has led to many challenges for both students and teachers [11]. Higher education students are particularly prone to mental health disorders during this pandemic being the high-risk age group to developing mental illness [12]. Even under normal circumstances, academic pressure and financial difficulties may cause anxiety and depression in university students [13].

The pandemic had a significant impact on higher education students’ practices regarding academic work and life, including the switch to online learning, closed libraries, altered communication channels for teachers’ and administrative support, new assessment methods, different workloads, and performance levels [14]. In addition, many students experienced disrupted social environments, with lockdown measures resulting in closed campuses, no meetings with friends, university colleagues, or relatives, no parties, no travelling, and remaining trapped abroad, etc. [15,16,17]. Many students had to face personal financial issues due to loss of student jobs and worries about their financial situation, future education, and career, resulting in emotional health issues, such as fears, frustrations, anxiety, anger, and boredom [18,19,20,21].

Mental health among university students is of significant public health concern, necessitating epidemiological data. Recent systematic reviews presenting evidence from primary research throughout the world emphasize the increased psychological cost linked with the COVID-19 pandemic. [22,23]. Most studies have reported an increase in students’ levels of stress/anxiety during the pandemic and the confinement period [24,25] along with six different themes of risk factors identified as psychological, academic, biological, lifestyle, social, and financial [26]. Women, younger kids, students with pre-existing health concerns, and those who spend at least one-third of their day on screens, and students with COVID-19-infected family or community members are at risk [26,27,28,29,30].

There is growing concern regarding university students’ mental health and the impact that undetected and untreated mental illness may have on students [31,32]. The abnormal stress and depression amongst students not only affects their performance but is also associated with heightened self-injury and suicidal attempts [24]. Chronic anxiety is associated with disability and lower academic achievement [31]. The severity of disability is further exacerbated when individuals enact avoidance behaviors characteristic amongst those with anxiety [33].

There have been several reports, opinion articles, and studies recently published on the psychological impact of the COVID-19 pandemic on college and medical students, but there is a gap in evidence synthesis on regional and global level estimates of anxiety/stress among university students during the pandemic [34,35,36,37,38,39]. There is a need to safeguard mental health and promote emotional and behavior balance in this population, through the implementation of evidence-based psychosocial interventions.

The aim of our review was to address this knowledge gap and conduct a quantitative systematic review to estimate the prevalence of anxiety in university students across continents during COVID-19. This systematic review will offer an insight into the anxiety prevalence among university students to aid decision-making and future research on mental health.

## 2. Materials and Methods

This systematic review and a subsequent meta-analysis were conducted in compliance with the recommendations and criteria described in the preferred reporting items for systematic reviews and meta-analyses (PRISMA).

### 2.1. Search Strategy

A comprehensive strategy was adopted to identify potential studies. First, a literature search was conducted through health-related research databases, including PubMed, Scopus, and PsycINFO. Search terms included were:

(a) (“students” OR “University students” OR “Post-secondary students” OR “graduate students”) AND
(b) (“severe acute respiratory syndrome coronavirus 2” OR “COVID-19” OR “SARS-CoV-2” OR “COVID-19 pandemic) AND
(c) (“Psychological Stress” OR “Anxiety” OR “stress”, “OR “Mental Health” OR “Mental Disorders” OR “Stress Disorders”).

**Figure 1:**
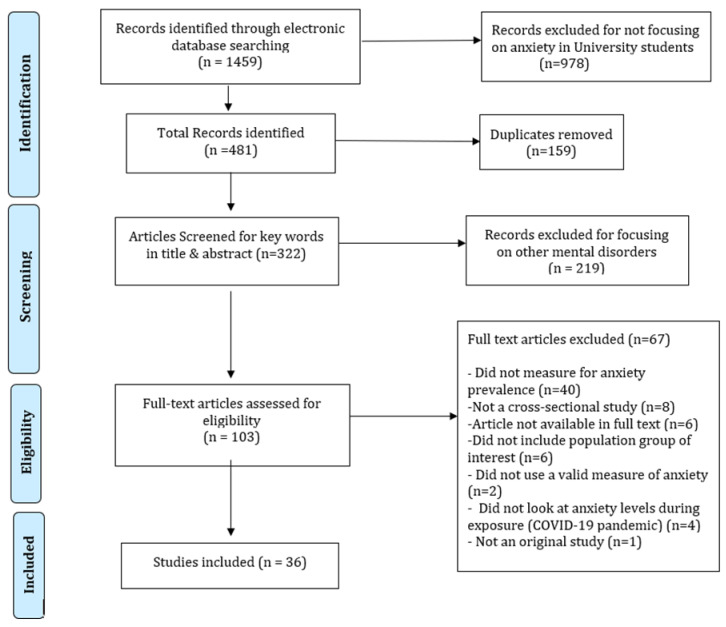
PRISMA flow chart.

The search period for relevant studies spanned from September 2020 to February 2021, with the last search conducted on February 17, 2021. The search process is shown in the flowchart (Figure 1). The search was conducted on titles, keywords, and abstracts with “AND” entered into the database search to link different categories (a, b, and c) of search terms. Truncation symbols (∗) were used to search for all possible forms of a search term (Table S1). We utilized forward reference searching to identify the references citing these articles and backward reference searching after reviewing the references that were cited in these articles.

### 2.2. Inclusion and Exclusion Criteria

The inclusion criteria were: [1] Cross-sectional studies reporting an anxiety condition related to COVID-19, in university or graduate students only, [2] using validated scales, [3] published until February 21, 2021, in peer-reviewed scientific journals [4] available in English. The exclusion criteria were: [1] studies focusing on mental disorders other than anxiety; [2] editorials, letter to the editors, viewpoints, case presentations, gray literature, or brief communications; and [3] population of interest was not university students.

All relevant studies were selected, the full-text versions were analyzed for methodological quality by three researchers (AK, TT, and WT) independently, and disagreement between reviewers was resolved by discussion or arbitration by the other researchers (SL and ZAB).

### 2.3. Data Retrieval

The data extraction included: author names, country of origin, objective, screening tools and cutoff values used for anxiety symptoms, and prevalence of anxiety symptoms (Table S2).

### 2.4. Outcome Measure

For the meta-analysis, the outcome measure was the prevalence of anxiety among university students during the COVID-19 pandemic.

### 2.5. Assessment of Quality for Included Studies

Before being included in the review, articles chosen for retrieval were examined for methodological validity by two independent reviewers (KS and SL) using the Joanna Briggs Institute (J.B.I.) standardized critical evaluation tool for prevalence studies [40].

### 2.6. Data Analysis

Since all the studies included were similar (cross-sectional studies), we conducted a proportion meta-analysis to assess the overall pooled prevalence of anxiety as an outcome and for assessment of heterogeneity. Data analysis was performed using the ‘R’ software R coding software and the RStudio visualization environment (R, R Core Team, Vienna, Austria).

### 2.7. Assessment of Heterogeneity

We used a random effects model to estimate the pooled prevalence and odds ratios (ORs) with a 95% confidence Interval (CI). Since a high heterogeneity across studies was expected, a random-effect model (DerSimonian-Laird) was considered, as opposed to the fixed effects model to adjust for the observed variability [41]. The Cochrane’s Q-test was used to assess heterogeneity at the significance level of *p* < 0.1. In addition, we used the I^2^ statistics to categorize heterogeneity as low (25–50%), moderate (51–75%), and high (above 75%) [42]. The results are presented in a forest plot with 95% confidence intervals (95% CI). In addition, subgroup analysis was performed for continents and gender-based prevalence of anxiety.

### 2.8. Publication Bias Assessment

To assess publication bias, the Egger’s test was conducted with the significance level of 0.05, and the corresponding Funnel plots were drawn. All statistical tests were two-sided.

### 2.9. Sensitivity Analysis

The leave-one-out method was used for the sensitivity analysis. At a time, one study was removed, and the pooled prevalence of the remaining studies were calculated to identify those single studies that could affect the pooled prevalence or heterogeneity.

## 3. Results

### 3.1. Selection and Evaluation of Studies

The search strategies using a combination of search terms identified a total of 1459 articles that included a search term from each category in their abstract or title (PRISMA flowchart). A total of 159 articles were duplicates. A database search was carried out by AK, TT, WT, and SL. Abstracts of 322 articles were reviewed by authors to do an initial screening of the eligibility for this systematic review. Out of these 322 articles, 219 were removed for not focusing specifically on anxiety. In total, 103 articles were selected for the full-text review, but 67 were excluded due to multiple reasons (Figure 1). Finally, a total sample of 36 studies were reviewed in full by two of the authors, including SL and KS. A mutual consensus was developed after final approval from ZAB.

### 3.2. Characteristics of the Included Studies

For the meta-analysis, a total of 36 studies that reported on the prevalence of anxiety in university students were included (Supplementary Materials File 1). All studies were of a cross-sectional design and had university students as the study population. The 21 studies included were from Asia including 11 from China, 2 from Bangladesh, 2 from Malaysia, 1 from Turkey, 1 from India, 1 from Nepal, 2 from Saudi Arabia, and 1 from Jordan. While nine studies were from Europe, five were from the USA and one from Egypt, Africa. A brief description of the characteristics of all included studies is shown in Table S2.

### 3.3. Screening Tools for Anxiety

Although similar in their methodological approach, different psychometric instruments were used in the studies. The instruments used to evaluate anxiety were: 7-item Generalized Anxiety Disorder Scale (GAD-7) [43,44,45,46,47,48,49,50,51,52,53,54,55,56,57,58,59]; 2-item Generalized Anxiety Disorder Scale (GAD-2) [60]; Self-Rating Anxiety Scale (SAS) [61,62,63,64,65,66,67,68]; Hamilton Anxiety Scale (HAM), Depression [52], Anxiety and Stress Scale (DASS-21) [34,69]; Patient health questionnaire (PHQ-4) [70,71]; State Trait Anxiety Inventory (STAI-Y2) [72]; and Health Anxiety Inventory (HAI) [73].

All studies were found to be conducted during a time period within the year 2020, even if the study was published within that year or 2021. The sampling was also different among these studies. Sample sizes varied amongst studies, with the smallest sample group being 105 [49] while the largest sample size was found to be 746,217 [45].

### 3.4. Quality Assessment

The risk of bias scores ranged from 5 to 8 out of a possible total of 9, with a mean score of 6.02 (Table S3). Most limitations within the studies were: (a) sampling method was non-random/convenience (instead of random sampling) or not clearly mentioned (20 studies), (b) sample size was not justified or calculated (22 studies), (c) coverage bias was present due to overrepresentation of subgroup within final responses (21 studies), or (d) the response rate was not justified or was inadequate (17 studies).

### 3.5. Prevalence of Anxiety

**Figure 2:**
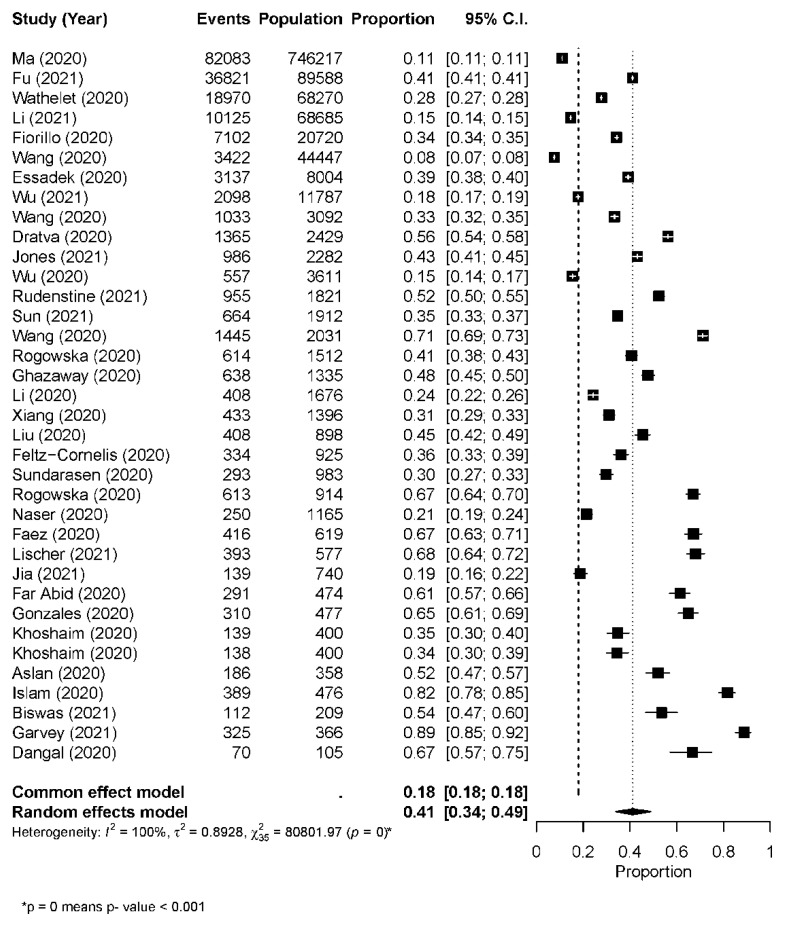
Forest plot of the prevalence of anxiety (all studies).

The overall pooled point estimates of the prevalence for anxiety varied between 11% and 89%, which were reported by 36 studies. All meta-analyses of the prevalence estimate of anxiety yielded a summary prevalence of 41% (95% CI = 0.34–0.49), with statistically significant evidence of between-study heterogeneity (Q = 80801.97, I2 = 100%, *p* ≤ 0.0001) (Figure 2).

### 3.6. Subgroup Analysis

In the subgroup analysis, 21 studies were from Asia, 9 studies were from Europe, and 5 studies were from the USA. A subgroup analysis based on continent-wise distribution of studies reported anxiety prevalence in Asia as 33% (95% CI:0.25–0.43), the prevalence of anxiety in Europe as 51% (95% CI: 0.44–0.59), and the highest prevalence of anxiety in the USA as 56% (95% CI: 0.44–0.67) (Figure S1).

Subgroup data by sex was reported by 17 studies for the prevalence of anxiety symptoms. A subgroup analysis based on sex reported a prevalence of anxiety in females as 43% (0.29–0.58) as compared to males with an anxiety prevalence of 39% (0.29–0.50) (Figure S2), but our results were insignificant due to overlapping confidence intervals.

### 3.7. Sensitivity Analysis

In the sensitivity analysis, when we excluded each study one-by-one from the analysis, it did not substantially change the pooled prevalence of anxiety. This indicates that no single study had a disproportionate impact on the overall prevalence. Sensitivity analysis indicated that no study influenced the pooled prevalence results by more than 2%.

### 3.8. Publication Bias

**Figure 3:**
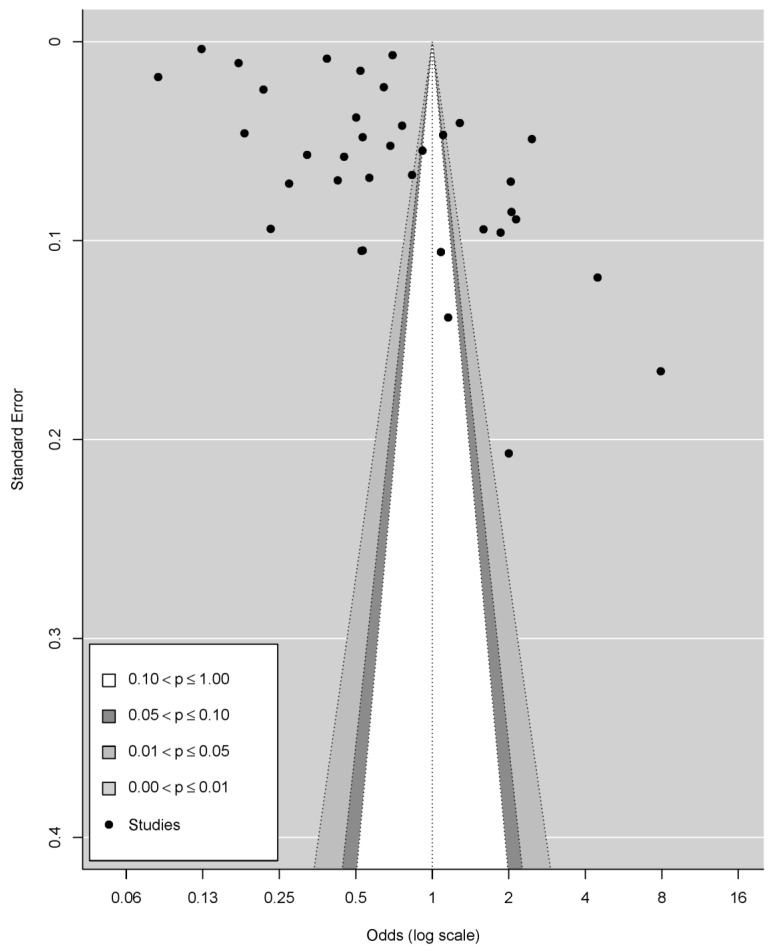
Funnel plot for publication bias.

Funnel plots indicated evidence of publication bias using visual inspection. To investigate publication bias in the retrieved articles, Egger’s test indices for the prevalence of anxiety were obtained, revealing that publication bias was significant (Figure 3).

## 4. Discussion

### 4.1. Overview and Interpretation of the Synthesized Findings

Anxiety disorders are the most prevalent mental health disorders and affect approximately one-third of adults during their lifetime [74,75]. According to American College Health Association, 2018, together with depression, they are the most commonly reported mental health disorder by university students and may significantly impact academic performance [76]. In 2017, WHO reported that the prevalence of anxiety and depression in the world population was estimated at 3.6% and 4.4%, respectively [77]. Using the parameters of the DSM-IV as criteria, research conducted by the WHO in 21 countries showed that 20.0% of university students had a mental disorder but only 16.4% of them received some type of treatment [78]. The mental health of university students during the COVID-19 pandemic is important as they have been identified as a particularly vulnerable population [79].

To our knowledge, this is the first quantitative review of the epidemiological burden of anxiety in university students throughout the world during the COVID-19 epidemic. We systematically identified 36 cross-sectional studies to quantitatively evaluate the pooled prevalence as well as estimating the prevalence in different sub-groups. Although the results of this review showed a prevalence of 41% (ranging from 11% to 89.0%) for anxiety in university students, meta-analytic models for anxiety revealed a high degree of publication bias. Therefore, the findings of our review remain inconclusive. The extreme range in estimates can be explained by the large heterogeneity of the sample, since distinct studies using the same scale have very different results. For example, among studies using the GAD-7 [43,44,48,50,52,53,54,55,56,58,59,72] and Zung SAS [61,62,64,65,66,67,68,80] scales, the prevalence ranges for symptoms of anxiety were 11–88.9% and 7.7–61.7%, respectively. This perhaps highlights the need to adapt the scales according to each country’s cultural context. Different scales and cut-off values can affect prevalence estimates, necessitating the use of validated scales with consistent cut-off values across populations. Each scale’s items and latent constructs must be re-evaluated in order for common scales with high reliability and validity to be widely utilized.

The COVID-19 pandemic poses a serious threat to global mental health, as evident by the available literature finding that mental health has been affected by this pandemic among different groups [13,22,23,37,81]. Our findings can be compared with reviews from different regions and population groups. For example, in a meta-analysis of 13 studies, anxiety and depression were found to be prevalent in 23.2% and 22.% of healthcare providers, respectively [13]. Our findings are consistent with prior systematic studies that found a higher prevalence of anxiety and depression in pandemic-affected college students, health workers, and nurses, implying a global mental health crisis linked to the epidemic [37,38,39]. Many students feel increased stress levels and anxiety symptoms because of changed delivery and uncertainty of university education, technological concerns of online courses, being far from home, social isolation, decreased family income, and future employment.

COVID-19 is having grave impacts on the mental health of university students. Lockdown, social isolation, and the disruption of daily life during the COVID-19 period have affected student lives and disposed them to stress, potentially creating a new public health crisis. Suicidal ideation is another serious mental disorder affecting university students significantly. Data from the WHO indicate that suicide was the second leading cause of death in the population between 15 and 29 years old, an age group commonly found in the university population [77]. There is some evidence of suicidal ideation among university students due to psychological pressures during COVID-19 [82]. Considering that mental health disorders, such as anxiety and depression, are strong predisposing factors for suicide [83,84], the high prevalence of these disorders found in this review may be associated with suicidal ideation among university students.

### 4.2. Prevalence of Anxiety by Geographical Regions

A subgroup analysis based on continent-wise distribution of studies reported anxiety prevalence in Asia as 33%, the prevalence of anxiety in Europe as 51%, and the highest prevalence of anxiety in the USA as 56%. In comparison with the infectious diseases literature, the scientific literature on mental health issues connected with the COVID-19 pandemic was scarce during the early months of the pandemic [85]. Furthermore, a large amount of the literature came from China and many western countries that were first hit by the pandemic. As a result, the findings of the available literature may not accurately reflect the true burden of anxiety in university students across continents during the later stages of the epidemic.

### 4.3. Subgroup Analyses (Sex)

In our review, female students were found to have a prevalence of anxiety symptoms of 43% compared to male students that had a prevalence of 39%, which is consistent with the available evidence, but our results were insignificant due to overlapping confidence intervals. Predictors of psychological distress included female gender, being a student, and poor physical health [13,21]. Gender and perceived vulnerability were found to have substantial impacts by Li et al. [86], with female students who believed themselves to be in danger being more prone to generalized and dissociated anxiety.

### 4.4. Future Research Directions

Our review synthesized evidence from cross-sectional studies with varying sample sizes. Future research should focus on large longitudinal studies that could provide more representative evidence on mental health among university students. The current evidence suggests the need for measures preventing the COVID-19 pandemic to address mental health disorders among university students. Keeping in view our findings, colleges and universities should develop policies and initiatives to address the root causes of students’ mental health problems. There is a need to address all aspects of well-being including physical, psychological, emotional, and social well-being. To prevent anxiety symptoms from evolving into more serious psychological problems, appropriate and regular mental health screening and initiatives should be designed including university counselling services, development of student mental health programs, as well as web-based programs for early screening and delivering interventions. The effectiveness of different interventions should be evaluated by adapting a comprehensive approach.

### 4.5. Limitations

This systematic review and meta-analysis were conducted using data from cross-sectional studies and have several limitations. First, we observed significant between-study heterogeneity that was not wholly accounted for in our subgroup analyses and sensitivity analyses. Additionally, it was difficult to incorporate the prevalence of depression and anxiety of included articles as they used different assessment tools. The assessment tools had a distinct standard, so the integrated results in our article only relatively revealed the true value. In the future, the results from different assessment tools should be integrated. Secondly, the lack of data in our subgroup analyses could have affected the effectiveness of our subgroup analysis. Therefore, we are not able to conclude with certainty that there was a difference observed in the prevalence of anxiety within continents or based on sex.

## 5. Conclusions

Mental health disorders are common disorders that have a significant prevalence among university students [78,87]. The results of this review highlight the importance of implementing strategies for prevention, intervention, and diagnosis/treatment of anxiety disorder among university students, who are a vulnerable group. There is a need to standardize different anxiety assessment tools in order to obtain integrated results from different populations. This could help in early identification and the design of early intervention to prevent serious mental illness among university students. Moreover, there is a need to develop effective risk communication strategies through, online and mass media health communication in collaboration with media partners and public health agencies.

## Supplementary Materials

The following are available online at https://www.mdpi.com/article/10.3390/ijerph19010062/s1, Table S1: Search strings for each data base. Table S2: Characteristics of included studies. Table S3: Quality assessment of studies based on Joanna Briggs Institute (JBI) standardized critical appraisal instrument for prevalence studies. Table S4: Assessment tools used by included studies to examine anxiety prevalence. Figure S1: Forest plot of anxiety prevalence by Continent (Panels A, B and C). Figure S2: Forest plot of anxiety prevalence by sex (Panels A and B). Supplementary Materials File 1: Studies included in the systematic review and Meta-analysis.
