# Supplementary material for: Prevalence of Anxiety in University Students during the COVID-19 Pandemic: A Systematic Review
Source: Int J Environ Res Public Health. 2021 Dec 22;19(1):62. doi: 10.3390/ijerph19010062 (PMC8750929; doi:10.3390/ijerph19010062)
Supplement: Supplementary file 1 [file ijerph-19-00062-s001.zip › ijerph-1471202-supplementary.pdf]

International Journal of
Environmental Research and Public Health

MDPI

Supplementary Material
Table S1: Search strings for each data base

Database	Search String
**PubMed** (Using the CATHD PubMed search string for COVID-19)	(university [tiab] OR college [tiab] OR postsecondary [tiab] OR post-secondary [tiab]) AND (students [tiab] OR students [mh:noexp] OR young adult [mesh]) AND (anxiety [tw] OR mental health [tiab] OR DASS-21 [tw] OR GAD-7 [tw] OR generalized anxiety disorder [tw] OR apprehension [tw] OR social anxiety [mh:noexp] OR social anxiety [tiab] OR anxieties [tw] OR anxiety disorders [tw:noexp] OR panic disorder [mh] OR fear [mh] OR panic [mh] OR sadness [mh] OR psychological distress [mh] OR loneliness [mh] OR activities of daily living [mh] OR stress,psychological [mh:noexp])AND (patient health questionnaire [mh] OR psychological tests [mh] OR surveys and questionnaires [mh:noexp] OR survey [tiab] OR Questionnaire [tiab] OR interview [tiab]) AND<br>((Coronavirus[mh:noexp] OR Betacoronavirus[mh:noexp] OR Coronavirus Infections[mh:noexp]) AND (Disease Outbreaks[mh:noexp] OR Epidemics[mh:noexp] OR Pandemics[mh])) OR COVID-19 diagnostic testing [Supplementary Concept] OR COVID-19 drug treatment [Supplementary Concept] OR COVID-19 serotherapy [Supplementary Concept] OR COVID-19 vaccine [Supplementary Concept] OR spike glycoprotein, COVID-19 virus [Supplementary Concept] OR COVID-19 [Supplementary Concept] OR severe acute respiratory syndrome coronavirus 2 [Supplementary Concept] OR nCoV[tiab] OR nCoV[tt] OR 2019nCoV[tiab] OR 2019nCoV[tt] OR 19nCoV[tiab] OR 19nCoV[tt] OR COVID19*[tiab] OR COVID19*[tt] OR COVID[tiab] OR COVID[tt] OR SARS-CoV-2[tiab] OR SARS-CoV-2[tt] OR SARSCOV-2[tiab] OR SARSCOV-2[tt] OR SARSCOV2[tiab] OR SARSCOV2[tt] OR Severe Acute Respiratory Syndrome Coronavirus 2[tiab] OR Severe Acute Respiratory Syndrome Coronavirus 2[tt] OR ((severe acute respiratory syndrome[tiab] OR severe acute respiratory syndrome[tt]) AND (corona virus 2[tiab] OR corona virus 2[tt])) OR new coronavirus[tiab] OR (new[tt] AND coronavirus[tt]) OR novel coronavirus[tiab] OR novel coronavirus[tt] OR novel corona virus[tiab] OR (novel[tt] AND corona virus[tt]) OR novel CoV[tiab] OR (novel[tt] AND CoV[tt]) OR novel HCoV[tiab] OR (novel[tt] AND HCoV[tt]) OR ((“19”[tiab] OR “19”[tt] OR “2019”[tiab] OR “2019”[tt] OR Wuhan[tiab] OR Wuhan[tt] OR Hubei[tiab] OR Hubei[tt]) AND (coronavirus*[tiab] OR coronavirus*[tt] OR corona virus*[tiab] OR corona virus*[tt] OR CoV[tiab] OR CoV[tt] OR HCoV[tiab] OR HCoV[tt])) OR ((coronavirus*[tiab] OR coronavirus*[tt] OR corona virus*[tiab] OR corona virus*[tt] OR betacoronavirus*[tiab] OR betacoronavirus*[tt]) AND (outbreak*[tiab] OR outbreak*[tt] OR epidemic*[tiab] OR epidemic*[tt] OR pandemic*[tiab] OR pandemic*[tt] OR crisis[tiab] OR crisis[tt])) OR ((Wuhan[tiab] OR Wuhan[tt] OR Hubei[tiab] OR Hubei[tt]) AND (pneumonia[tiab] OR pneumonia[tt]))<br>AND 2019/10/31:3000/12/31[Date – Publication]

**PsycINFO**	students OR Any Field: "Young adult*" AND Any Field: college* OR Any Field: "post-secondary" OR Any Field: postsecondary OR Any Field: universit* AND Any Field: coronavirus OR Any Field: "COVID-19" AND Any Field: "Mental Health" OR Any Field: "Generalized Anxiety" OR Any Field: disorder OR Any Field: "Anxiety" OR Any Field: stress
**Scopus**	(TITLE-ABS-KEY (college* OR "post-secondary" OR postsecondary OR universit*) AND TITLE-ABS-KEY (students OR "Young adult*") AND TITLE-ABS-KEY (coronavirus OR "COVID-19") AND TITLE-ABS-KEY ("Psychological impact" OR "Mental health" OR "DASS-21" OR "GAD-7" OR apprehension OR anxiet*) AND TITLE-ABS-KEY (survey OR questionnaire OR interview))

Table S2: Characteristics of included studies

Sr. No.	Authors	Year	Country	Assessment Tools	Cut off value for anxiety tools	Objective of the studies	Anxiety Prevalence N (%)
1.	Aslan et al.	2020	Turkey	GAD-7	Scores > 10	To examine the prevalence of perceived stress and mental health among students during the pandemic	186 (52%)
2.	Biswas et al.	2021	India	GAD-7, HAM	HAM-A score >0 GAD-7 score >7	To evaluate anxiety level among students	112 (53.87%)
3.	Dangal et al.	2020	Nepal	GAD-7	N/A	To find out the psychological impacts of COVID-19 on students	70 (66.7%)
4.	Dratva et al.	2020	Switzerland	GAD-7	Score >7	To evaluate the mental situation of students during the epidemic	1365(61.4%)
5.	Essadek et al.	2020	France	GAD-7	Score ≥ 7	To evaluate the impact of Covid-19 on the mental health	3137(39.19%)
6.	Faez et al.	2020	Malaysia	DASS-21	N/A	To explore the impact of lockdown due to COVID-19 on mental health	416(67.21%)
7.	Far Abid et al.	2020	Bangladesh	Zung's SAS	Score >45	To investigate the socio-psychological impact on students during COVID-19	291(61.07%)
8.	Fiorillo et al.	2020	Italy	DASS-21	Score >6	To examine level of anxiety, depression during COVID-19	7102(34.3%)
9.	Fu et al.	2021	China	GAD-7	Score >4	To examine the mental health of students during the COVID-19	36821(41.1%)
10.	Garvey et al.	2021	Spain	GAD-7	N/A	To measure the effect that a decrease in students' quality of life has on anxiety levels	325(88.9%)
11.	Ghazawy et al.	2020	Egypt	DASS-21	Score >7	To investigate the psychological impacts on university students	638(53.6%)
12.	Gonzales et al.	2020	USA	GAD-2	N/A	To examine the mental health needs of college students	310(65%)
13.	Islam et al.	2020	Bangladesh	GAD-7	Score >5	To investigate the prevalence of depression and anxiety amongst university students	389(87.7%)
14.	Jia et al.	2021	China	Zung's SAS	Score ≥50	To investigate the knowledge-attitude-practice (KAP) of students	139(18.78%)
15.	Jones et al.	2021	USA	PHQ-4	N/A	To investigate the impact of the COVID-19 on student's mental health	986(43.2%)
16.	Khoshaim et al.	2020	Saudi Arabia	Zung's SAS	Score ≥45	To assess the anxiety status of students	138(34.6%)
17.	Khoshaim et al.	2020	Saudi Arabia	Zung's SAS	Score ≥45	To determine the coping strategies by university students	139(34.9%)
18.	Li et al.	2020	China	HAI	Score ≥15	To explore the prevalence of panic and anxiety among students	408(24.3%)
19.	Li et al.	2021	China	GAD-7	Score ≥ 7	To describe trajectory changes of acute stress, anxiety and depressive symptoms from the early phase to under control phase	10125(14.7%)

20.	Lischer et al.	2021	Switzerland	PHQ-4	Score ≥ 4	To investigate the mental health status of university students	393(85.8%)
21.	Liu et al.	2020	USA	GAD-7	Score ≥ 10	Identify salient psychosocial risks for mental health symptoms	408(45.4%)
22.	Ma et al.	2020	China	GAD-7	Score ≥7	To assess the mental health problems and epidemiological characteristics	82083(11%)
23.	Naser et al.	2020	Jordan	PHQ-9, GAD-7	GAD-Score ≥ 5	To explore the prevalence of depression and anxiety among university students	250(21.5%)
24.	Rogowska et al.	2020	Poland	GAD-7	Score ≥ 5	To examine the association of anxiety with self-rated general health, satisfaction with life, stress and coming strategies of students	613(65%)
25.	Rogowska et al.	2020	Ukraine	GAD-7	Score ≥ 5	To examine the relationship between physical activity and the mental health of students	614(59.13%)
26.	Rudenstine et al.	2020	USA	GAD-7	Score ≥ 5	To document the stressors experienced consequent to the COVID-19 pandemic	955(73.4%)
27.	Sun et al.	2021	China	GAD-7	Score ≥ 5	To investigate the prevalence of mental health issues among university students	664(34.73%)
28.	Sundarasen et al.	2020	Malaysia	Zung's SAS	Score ≥45	To evaluate the impacts of COVID-19 ad lockdown on socio-psychological well-being and anxiety	293(29.8%)
29.	Van Der Feltz-Cornelis et al.	2020	United Kingdom	GAD-7	Score ≥ 5	To explore how COVID-19 and arrangements like remote working affect work or study stress levels, and functioning	334(37.2%)
30.	Wang et al	2020	China	GAD-7	Score ≥ 5	To explore the psychological situation and influence of the outbreak on college students	1033(33.4%)
31.	Wang et al.	2020	USA	GAD-7	Score ≥ 5	To assess the mental health among college students during the pandemic	1445(71.75%)
32.	Wang et al.	2020	China	Zung's SAS	Score ≥50	To explore the prevalence of anxiety and depression symptoms among college students	3422(7.7%)
33.	Wathelet et al.	2020	France	STAI Y-2	Score ≥46	To measure the prevalence of self-reported mental health symptoms	18970(27.5%)
34.	Wu et al.	2021	China	Zung's SAS	Score ≥50	To evaluate and analyze student's anxiety in university students	557(30.75%)
35.	Wu et al.	2021	China	GAD-7	Score ≥ 5	To estimate the prevalence of anxiety and depressive symptoms among college students	2098(17.8%)
36.	Xiang et al.	2020	China	Zung's SAS	Score ≥50	To evaluate the prevalence of inadequate physical activity, anxiety and depression among college students	433(31%)

International Journal of Environmental Research and Public Health

Table S3: Quality assessment of studies based on Joanna Briggs Institute (JBI) standardized critical appraisal instrument for prevalence studies

Study	Q1	Q2	Q3	Q4	Q5	Q6	Q7	Q8	Q9	Total Score
Aslan et al.	Y	N	Y	Y	N	Y	Y	Y	N	5
Biswas et al.	Y	N	N	Y	N	Y	Y	Y	N	5
Dangal et al.	Y	N	N	Y	N	Y	Y	Y	Y	6
Dratva et al.	Y	N	N	Y	N	Y	Y	Y	N	5
Essadek et al.	Y	Y	N	Y	Y	Y	Y	Y	N	7
Faez et al.	Y	N	Y	Y	N	Y	Y	Y	Y	7
Far Abid et al.	Y	Y	N	Y	N	Y	Y	Y	N	6
Fiorillo et al.	Y	N	N	Y	N	Y	Y	Y	N	5
Fu et al.	Y	N	N	Y	N	Y	Y	Y	N	5
Garvey et al.	Y	N	N	Y	N	Y	Y	Y	N	5
Ghazawy et al.	Y	N	N	Y	N	Y	Y	Y	N	5
Gonzales et al.	Y	N	N	Y	N	Y	Y	Y	N	5
Islam et al.	Y	N	N	Y	N	Y	Y	Y	N	5
Jia et al.	Y	N	N	Y	N	Y	Y	Y	Y	6
Jones et al.	Y	Y	N	Y	Y	Y	Y	Y	Y	8
Khoshaim et al.	Y	N	N	Y	N	Y	Y	Y	Y	6
Khoshaim et al.	Y	N	Y	Y	N	Y	Y	Y	N	6
Li et al.	Y	N	N	Y	N	Y	Y	Y	N	5
Li et al.	Y	Y	N	Y	N	Y	Y	Y	N	5
Lischer et al.	Y	N	N	Y	Y	Y	Y	Y	Y	7
Liu et al.	Y	N	N	Y	N	Y	Y	Y	N	5
Ma et al.	Y	Y	N	Y	N	Y	Y	Y	Y	7
Naser et al.	Y	N	Y	Y	N	Y	Y	Y	N	6
Rogowska et al.	Y	Y	N	Y	N	Y	Y	Y	Y	7
Rogowska et al.	Y	N	N	Y	Y	Y	Y	Y	Y	7
Rudenstine et al.	Y	N	N	Y	Y	Y	Y	Y	N	6
Sun et al.	Y	N	N	Y	N	Y	Y	Y	N	5
Sundarasen et al.	Y	N	N	Y	N	Y	Y	Y	N	5
Van DerFeltz- et al.	Y	Y	N	Y	Y	Y	Y	Y	Y	8
Wang et al	Y	Y	N	Y	N	Y	Y	Y	N	6
Wang et al.	Y	Y	N	Y	Y	Y	Y	Y	Y	8
Wang et al	Y	Y	N	Y	Y	Y	Y	Y	Y	8
Wathelet et al	Y	Y	N	Y	N	Y	Y	Y	Y	7

Wu et al.	Y	N	N	Y	N	Y	Y	Y	N	5
Wu, 2021	Y	Y	N	Y	Y	Y	Y	Y	Y	8
Xiang, 2020	Y	N	N	Y	N	Y	Y	Y	N	5

Note: Question for Risk of bias are labeled from 1-10. Answers are recorded as follows. N:No; Y:Yes;1: Was the sample frame appropriate to address the target population?; 2: Were study participants recruited in an appropriate way?; 3: Was the sample size adequate?; 4: Were the study subjects and setting described in detail?; 5: Was data analysis conducted with sufficient coverage of the identified sample?; 6: Were valid methods used for the identification of the condition?; 7: Was the condition measured in a standard, reliable way for all participants?; 8: Was there appropriate statistical analysis?; 9: Was the response rate adequate, and if not, was the low response rate managed appropriately?. Score of quality was based on the Joanna Briggs Institute (JBI) standardized critical appraisal instrument for prevalence studies

Table S4: Assessment tools used by included studies to examine anxiety prevalence

**Assessment Tools for Anxiety**	**Assessment Tools (abbreviated)**	**Standard**	**Studies (n)**
Generalized Anxiety Disorder (2-item)	GAD-2	A score of ≥3 out of 6 indicates anxiety.	1
State-Trait Anxiety Inventory	STAI Y-2	A score of <46 out of 80 indicates low anxiety. A score >46 out of 80 indicates moderate to severe anxiety.	1
Generalized Anxiety Disorder (7-item)	GAD-7	A score of ≥5 out of 21 indicates anxiety	20
Hamilton Anxiety Rating Scale	HAM-A	A score of ≥7 out of 30 indicates anxiety	1
Depression, Anxiety and Stress Scale 21-items	DASS-21	A score of ≥8 out of 42 indicates anxiety	3
Zung's Self-Rated Anxiety Scale	SAS	A score of ≥50 out of 80 indicates anxiety	8
Patient Health Questionnaire-4	PHQ-4	A score of ≥4 out of 12 indicates anxiety	2
Health Anxiety Inventory	HAI	A score of ≥15 out of 18 indicates anxiety	1

Supplementary Materials File 1: Studies included in the systematic review & Meta- analysis

1. Akhtarul Islam, M., Barna, S. D., Raihan, H., Nafiul Alam Khan, M., & Tanvir Hossain, M. (2020). Depression and anxiety among university students during the COVID-19 pandemic in Bangladesh: A web-based cross-sectional survey. In *PLoS ONE* (Vol. 15, Issue 8 August, p. e0238162). Public Library of Science. https://doi.org/10.1371/journal.pone.0238162
2. Aslan, I., Ochnik, D., & Çınar, O. (2020). Exploring perceived stress among students in Turkey during the covid-19 pandemic. *International Journal of Environmental Research and Public Health*, *17*(23), 1–17. https://doi.org/10.3390/ijerph17238961
3. Biswas, S., & Biswas, A. (2021). Anxiety level among students of different college and universities in India during lock down in connection to the COVID-19 pandemic. *Journal of Public Health (Germany)*, 1–7. https://doi.org/10.1007/s10389-020-01431-8
4. D'Hondt, F., Wathelet, M., Duhem, S., Vaiva, G., Baubet, T., Habran, E., Veerapa, E., Debien, C., Molenda, S., Horn, M., Grandgenèvre, P., Notredame, C. E., & D'Hondt, F. (2020). Factors Associated with Mental Health Disorders among University Students in France Confined during the COVID-19 Pandemic. *JAMA Network Open*, *3*(10). https://doi.org/10.1001/jamanetworkopen.2020.25591
5. Dratva, J., Zysset, A., Schlatter, N., von Wyl, A., Huber, M., & Volken, T. (2020). Swiss university students' risk perception and general anxiety during the covid-19 pandemic. *International Journal of Environmental Research and Public Health*, *17*(20), 1–11. https://doi.org/10.3390/ijerph17207433
6. Essadek, A., & Rabeyron, T. (2020). Mental health of French students during the Covid-19 pandemic. In *Journal of Affective Disorders* (Vol. 277, pp. 392–393). Elsevier B.V. https://doi.org/10.1016/j.jad.2020.08.042
7. Faez, M., Hadi, J., Abdalqader, M., Assem, H., Ads, H. O., & Ghazi, H. F. (n.d.). Impact of Lockdown Due to Covid-19 on Mental Health among Students in Private University at Selangor. In *European Journal of Molecular & Clinical Medicine* (Vol. 7, Issue 11).
8. Far Abid Hossain, S., Nurunnabi, M., Sundarasen, S., Chinna, K., Kamaludin, K., Baloch, G. M., Khoshaim, H. B., & Sukayt, A. (2021). Socio-psychological impact on Bangladeshi students during COVID-19. *Journal of Public Health Research*, *9*(s1), 38–44. https://doi.org/10.4081/jphr.2020.1911
9. Fiorillo, A., Sampogna, G., Giallonardo, V., Del Vecchio, V., Luciano, M., Albert, U., Carmassi, C., Carrà, G., Cirulli, F., Dell'Osso, B., Nanni, M. G., Pompili, M., Sani, G., Tortorella, A., & Volpe, U. (2020). Effects of the lockdown on the mental health of the general population during the COVID-19 pandemic in Italy: Results from the COMET collaborative network. *European Psychiatry*, *63*(1). https://doi.org/10.1192/j.eurpsy.2020.89
10. Fu, W., Yan, S., Zong, Q., Anderson-Luxford, D., Song, X., Lv, Z., & Lv, C. (2021). Mental health of college students during the COVID-19 epidemic in China. *Journal of Affective Disorders*, *280*, 7–10. https://doi.org/10.1016/j.jad.2020.11.032
11. Garvey, A. M., García, I. J., Franco, S. H. O., & Fernández, C. M. (2021). The psychological impact of strict and prolonged confinement on business students during the COVID-19 pandemic at a Spanish university. *International Journal of Environmental Research and Public Health*, *18*(4), 1–13. https://doi.org/10.3390/ijerph18041710
12. Ghazawy, E. R., Ewis, A. A., Mahfouz, E. M., Khalil, D. M., Arafa, A., Mohammed, Z., Mohammed, E.-N. F., Hassan, E. E., Abdel Hamid, S., Ewis, S. A., & Mohammed, A. E.-N. S. (2020). Psychological impacts of COVID-19 pandemic on the university students in Egypt. *Health Promotion International*. https://doi.org/10.1093/heapro/daaa147
13. Gonzales, G., Loret de Mola, E., Gavulic, K. A., McKay, T., & Purcell, C. (2020). Mental Health Needs Among Lesbian, Gay, Bisexual, and Transgender College Students During the COVID-19 Pandemic. *Journal of Adolescent Health*, *67*(5), 645–648. https://doi.org/10.1016/j.jadohealth.2020.08.006
14. Jia, Y., Qi, Y., Bai, L., Han, Y., Xie, Z., & Ge, J. (2021). Knowledge-attitude-practice and psychological status of college students during the early stage of COVID-19 outbreak in China: a cross-sectional study. *BMJ Open*, *11*, 45034. https://doi.org/10.1136/bmjopen-2020-045034
15. Jones, H. E., Manze, M., Ngo, V., Lamberson, P., & Freudenberg, N. (2021). The Impact of the COVID-19 Pandemic on College Students' Health and Financial Stability in New York City: Findings from a Population-Based Sample of City University of New York (CUNY) Students. *Journal of Urban Health*, *98*(2), 187–196. https://doi.org/10.1007/s11524-020-00506-x

16. Khoshaim, H. B., Al-Sukayt, A., Chinna, K., Nurunnabi, M., Sundarasen, S., Kamaludin, K., Baloch, G. M., & Hossain, S. F. A. (2020). How students in the Kingdom of Saudi Arabia are coping with COVID-pandemic 19. *Journal of Public Health Research*, *9*(S1), 17–23. https://doi.org/10.4081/jphr.2020.1898
17. Khoshaim, H. B., Al-Sukayt, A., Chinna, K., Nurunnabi, M., Sundarasen, S., Kamaludin, K., Baloch, G. M., & Hossain, S. F. A. (2020). Anxiety Level of University Students During COVID-19 in Saudi Arabia. *Frontiers in Psychiatry*, *11*, 579750. https://doi.org/10.3389/fpsyt.2020.579750
18. Li, M., Liu, L., Yang, Y., Wang, Y., Yang, X., & Wu, H. (2020). Psychological impact of health risk communication and social media on college students during the covid-19 pandemic: Cross-sectional study. *Journal of Medical Internet Research*, *22*(11), e20656. https://doi.org/10.2196/20656
19. Li, Y., Zhao, J., Ma, Z., McReynolds, L. S., Lin, D., Chen, Z., Wang, T., Wang, D., Zhang, Y., Zhang, J., Fan, F., & Liu, X. (2021). Mental Health Among College Students During the COVID-19 Pandemic in China: A 2-Wave Longitudinal Survey. *Journal of Affective Disorders*, *281*, 597–604. https://doi.org/10.1016/j.jad.2020.11.109
20. Lischer, S., Safi, N., & Dickson, C. (2021). Remote learning and students' mental health during the Covid-19 pandemic: A mixed-method enquiry. *Prospects*, 1. https://doi.org/10.1007/s11125-020-09530-w
21. Liu, C. H., Zhang, E., Wong, G. T. F., Hyun, S., & Hahm, H. "Chris." (2020). Factors associated with depression, anxiety, and PTSD symptomatology during the COVID-19 pandemic: Clinical implications for U.S. young adult mental health. *Psychiatry Research*, *290*, 113172. https://doi.org/10.1016/j.psychres.2020.113172
22. Ma, Z., Zhao, J., Li, Y., Chen, D., Wang, T., Zhang, Z., Chen, Z., Yu, Q., Jiang, J., Fan, F., & Liu, X. (2020). Mental health problems and correlates among 746 217 college students during the coronavirus disease 2019 outbreak in China. *Epidemiology and Psychiatric Sciences*, *29*. https://doi.org/10.1017/S2045796020000931
23. Naser, A. Y., Dahmash, E. Z., Al-Rousan, R., Alwafi, H., Alrawashdeh, H. M., Ghoul, I., Abidine, A., Bokhary, M. A., AL-Hadithi, H. T., Ali, D., Abuthawabeh, R., Abdelwahab, G. M., Alhartani, Y. J., Al Muhaisen, H., Dagash, A., & Alyami, H. S. (2020). Mental health status of the general population, healthcare professionals, and university students during 2019 coronavirus disease outbreak in Jordan: A cross-sectional study. *Brain and Behavior*, *10*(8). https://doi.org/10.1002/brb3.1730
24. Raj Dangal, M. (2020). 2| ISSUE 70 |COVID-19 SPECIAL ISSUE| 2020 Citation Dangal MR, Bajracharya LS. Students Anxiety Experiences during COVID-19 in Nepal. In *Kathmandu Univ Med J* (Vol. 18, Issue 2).
25. Rogowska, A. M., Pavlova, I., Kuśnierz, C., Ochnik, D., Bodnar, I., & Petrytsa, P. (2020). Does Physical Activity Matter for the Mental Health of University Students during the COVID-19 Pandemic? *Journal of Clinical Medicine*, *9*(11), 3494. https://doi.org/10.3390/jcm9113494
26. Rogowska, A. M., Kuśnierz, C., & Bokszczanin, A. (2020). Examining anxiety, life satisfaction, general health, stress and coping styles during COVID-19 pandemic in Polish sample of university students. *Psychology Research and Behavior Management*, *13*, 797.
27. Rudenstine, S., McNeal, K., Schulder, T., Ettman, C. K., Hernandez, M., Gvozdieva, K., & Galea, S. (2021). Depression and Anxiety During the COVID-19 Pandemic in an Urban, Low-Income Public University Sample. *Journal of Traumatic Stress*, *34*(1), 12–22. https://doi.org/10.1002/jts.22600
28. Sun, S., Goldberg, S. B., Lin, D., Qiao, S., & Operario, D. (2021). Psychiatric symptoms, risk, and protective factors among university students in quarantine during the COVID-19 pandemic in China. *Globalization and Health*, *17*(1), 1–14. https://doi.org/10.1186/s12992-021-00663-x
29. Sundarasen, S., Chinna, K., Kamaludin, K., Nurunnabi, M., Baloch, G. M., Khoshaim, H. B., Hossain, S. F. A., & Sukayt, A. (2020). Psychological impact of covid-19 and lockdown among university students in malaysia: Implications and policy recommendations. *International Journal of Environmental Research and Public Health*, *17*(17), 1–13. https://doi.org/10.3390/ijerph17176206

30. Van Der Feltz-Cornelis, C. M., Varley, D., Allgar, V. L., & de Beurs, E. (2020). Workplace Stress, Presenteeism, Absenteeism, and Resilience Amongst University Staff and Students in the COVID-19 Lockdown. *Frontiers in Psychiatry*, *11*, 588803. https://doi.org/10.3389/fpsyt.2020.588803
31. Wang, C., & Zhao, H. (2020). The Impact of COVID-19 on Anxiety in Chinese University Students. *Frontiers in Psychology*, *11*. https://doi.org/10.3389/fpsyg.2020.01168
32. Wang, X., Hegde, S., Son, C., Keller, B., Smith, A., & Sasangohar, F. (2020). Investigating mental health of US college students during the COVID-19 pandemic: Cross-sectional survey study. *Journal of Medical Internet Research*, *22*(9). https://doi.org/10.2196/22817
33. Wang, X., Chen, H., Liu, L., Liu, Y., Zhang, N., Sun, Z., Lou, Q., Ge, W., Hu, B., & Li, M. (2020). Anxiety and Sleep Problems of College Students During the Outbreak of COVID-19. *Frontiers in Psychiatry*, *11*, 588693. https://doi.org/10.3389/fpsyt.2020.588693
34. Wang, Z. H., Yang, H. L., Yang, Y. Q., Liu, D., Li, Z. H., Zhang, X. R., Zhang, Y. J., Shen, D., Chen, P. L., Song, W. Q., Wang, X. M., Wu, X. B., Yang, X. F., & Mao, C. (2020). Prevalence of anxiety and depression symptom, and the demands for psychological knowledge and interventions in college students during COVID-19 epidemic: A large cross-sectional study. *Journal of Affective Disorders*, *275*, 188–193. https://doi.org/10.1016/j.jad.2020.06.034
35. Wu, X., Tao, S., Zhang, Y., Li, S., Ma, L., Yu, Y., Sun, G., Li, T., & Tao, F. (2021). Geographic distribution of mental health problems among Chinese college students during the COVID-19 Pandemic: Nationwide, web-based survey study. *Journal of Medical Internet Research*, *23*(1). https://doi.org/10.2196/23126
36. Xiang, M. Q., Tan, X. M., Sun, J., Yang, H. Y., Zhao, X. P., Liu, L., Hou, X. H., & Hu, M. (2020). Relationship of Physical Activity with Anxiety and Depression Symptoms in Chinese College Students During the COVID-19 Outbreak. *Frontiers in Psychology*, *11*. https://doi.org/10.3389/fpsyg.2020.582436

Figure S1: Forest plot of anxiety prevalence by Continent (Panels A, B and C)

PANEL A

Study (Year)	Events	Population	Proportion	95% C.I.
Ma (2020)	82083	746217	0.11	[0.11; 0.11]
Fu (2021)	36821	89588	0.41	[0.41; 0.41]
Li (2021)	10125	68685	0.15	[0.14; 0.15]
Wang (2020)	3422	44447	0.08	[0.07; 0.08]
Wu (2021)	2098	11787	0.18	[0.17; 0.19]
Wang (2020)	1033	3092	0.33	[0.32; 0.35]
Wu (2020)	557	3611	0.15	[0.14; 0.17]
Sun (2021)	664	1912	0.35	[0.33; 0.37]
Li (2020)	408	1676	0.24	[0.22; 0.26]
Xiang (2020)	433	1396	0.31	[0.29; 0.33]
Sundarasen (2020)	293	983	0.30	[0.27; 0.33]
Naser (2020)	250	1165	0.21	[0.19; 0.24]
Faez (2020)	416	619	0.67	[0.63; 0.71]
Jia (2021)	139	740	0.19	[0.16; 0.22]
Far Abid (2020)	291	474	0.61	[0.57; 0.66]
Khoshaim (2020)	139	400	0.35	[0.30; 0.40]
Khoshaim (2020)	138	400	0.34	[0.30; 0.39]
Aslan (2020)	186	358	0.52	[0.47; 0.57]
Islam (2020)	389	476	0.82	[0.78; 0.85]
Biswas (2021)	112	209	0.54	[0.47; 0.60]
Dangal (2020)	70	105	0.67	[0.57; 0.75]
**Common effect model**		.	**0.16**	**[0.15; 0.16]**
**Random effects model**			**0.33**	**[0.25; 0.43]**

Heterogeneity: $I^2 = 100\%$, $\tau^2 = 0.9633$, $\chi^2_{20} = 56685.83$ (p = 0)*

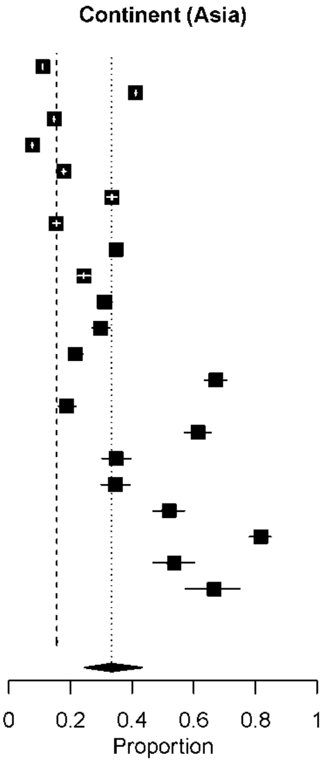

*p = 0 means p- value < 0.001

PANEL B

Study (Year)	Events	Population	Proportion	95% C.I.
Wathelet (2020)	18970	68270	0.28	[0.27; 0.28]
Fiorillo (2020)	7102	20720	0.34	[0.34; 0.35]
Essadek (2020)	3137	8004	0.39	[0.38; 0.40]
Dratva (2020)	1365	2429	0.56	[0.54; 0.58]
Rogowska (2020)	614	1512	0.41	[0.38; 0.43]
Feltz-Cornelis (2020)	334	925	0.36	[0.33; 0.39]
Rogowska (2020)	613	914	0.67	[0.64; 0.70]
Lischer (2021)	393	577	0.68	[0.64; 0.72]
Garvey (2021)	325	366	0.89	[0.85; 0.92]
**Common effect model**		.	**0.32**	**[0.31; 0.32]**
**Random effects model**			**0.51**	**[0.44; 0.59]**

Heterogeneity: $I^2 = 100\%$, $\tau^2 = 0.2039$, $\chi^2_{8} = 2458.38$ (p = 0)*

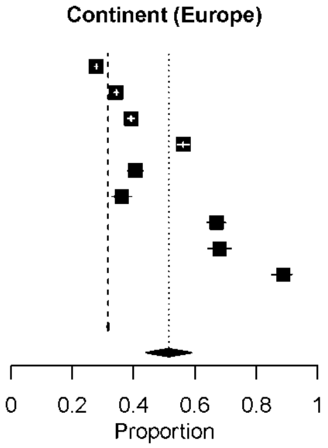

*p = 0 means p- value < 0.001

PANEL C

Study (Year)	Events	Population	Proportion	95% C.I.
Jones (2021)	986	2282	0.43	[0.41; 0.45]
Rudenstine (2021)	955	1821	0.52	[0.50; 0.55]
Wang (2020)	1445	2031	0.71	[0.69; 0.73]
Liu (2020)	408	898	0.45	[0.42; 0.49]
Gonzales (2020)	310	477	0.65	[0.61; 0.69]
**Common effect model**		.	**0.54**	**[0.53; 0.55]**
**Random effects model**			**0.56**	**[0.44; 0.67]**

Heterogeneity: $I^2 = 99\%$, $\tau^2 = 0.2862$, $\chi^2_4 = 385.76$ ($p < 0.01$)

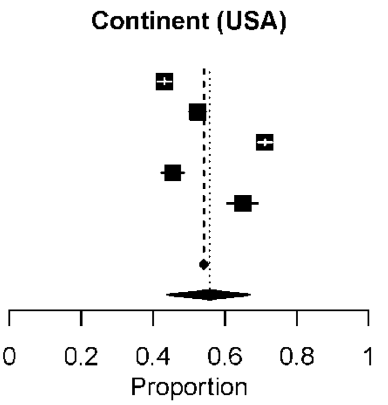

Figure S2: Forest plot of anxiety prevalence by sex (Panels A and B)

PANEL A

Study (Year)	Events	Population	Proportion	95% C.I.
Fu (2021)	16028	23166	0.69	[0.69; 0.70]
Wathelet (2020)	6142	11877	0.52	[0.51; 0.53]
Wu (2021)	784	4272	0.18	[0.17; 0.20]
Essadek (2020)	702	1902	0.37	[0.35; 0.39]
Wu (2020)	220	1234	0.18	[0.16; 0.20]
Wang (2020)	169	869	0.19	[0.17; 0.22]
Li (2020)	146	442	0.33	[0.29; 0.38]
Rudenstine (2021)	162	331	0.49	[0.44; 0.54]
Islam (2020)	62	258	0.24	[0.19; 0.30]
Jia (2021)	42	240	0.17	[0.13; 0.23]
Khoshaim (2020)	23	76	0.30	[0.21; 0.41]
Sundarasen (2020)	16	314	0.05	[0.03; 0.08]
Dangal (2020)	19	41	0.46	[0.32; 0.61]
Garvey (2021)	14	51	0.27	[0.17; 0.41]
Far Abid (2020)	142	151	0.94	[0.89; 0.97]
Biswas (2021)	18	26	0.69	[0.49; 0.84]
Gonzales (2020)	36	38	0.95	[0.81; 0.99]
**Common effect model**		.	**0.56**	**[0.55; 0.56]**
**Random effects model**			**0.39**	**[0.29; 0.50]**

Heterogeneity: I^2 = 100%, τ^2 = 0.8745, χ^2_{16} = 5367.16 (p = 0)*

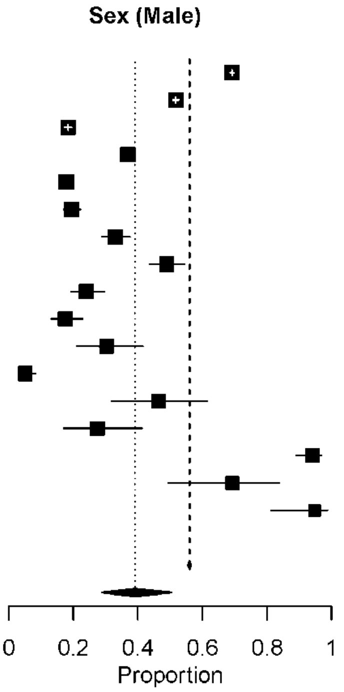

*p = 0 means p- value < 0.001

PANEL B

Study (Year)	Events	Population	Proportion	95% C.I.
Fu (2021)	20873	29557	0.71	[0.70; 0.71]
Wathelet (2020)	23354	26897	0.87	[0.86; 0.87]
Wu (2021)	1314	5417	0.24	[0.23; 0.25]
Essadek (2020)	2435	2965	0.82	[0.81; 0.83]
Wang (2020)	351	1703	0.21	[0.19; 0.23]
Wu (2020)	337	1820	0.19	[0.17; 0.20]
Li (2020)	262	826	0.32	[0.29; 0.35]
Rudenstine (2021)	572	729	0.78	[0.75; 0.81]
Jia (2021)	97	361	0.27	[0.23; 0.32]
Sundarasen (2020)	63	590	0.11	[0.08; 0.13]
Biswas (2021)	93	183	0.51	[0.44; 0.58]
Khoshaim (2020)	115	186	0.62	[0.55; 0.69]
Gonzales (2020)	75	127	0.59	[0.50; 0.67]
Far Abid (2020)	41	140	0.29	[0.22; 0.37]
Islam (2020)	25	131	0.19	[0.13; 0.27]
Dangal (2020)	51	64	0.80	[0.68; 0.88]
Garvey (2021)	8	125	0.06	[0.03; 0.12]
**Common effect model**		.	**0.70**	**[0.69; 0.70]**
**Random effects model**			**0.43**	**[0.29; 0.58]**

Heterogeneity: I^2 = 100%, τ^2 = 1.5341, χ^2_{16} = 11837.02 (p = 0)*

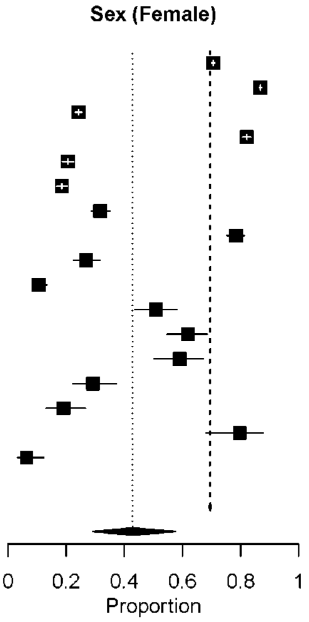

*p = 0 means p- value < 0.001
